# Safety and feasibility of same-day discharge following lumbar decompression surgery: A systematic review

**DOI:** 10.1016/j.bas.2022.100888

**Published:** 2022-04-18

**Authors:** Edward Goacher, Matthew I. Sanders, Marcel Ivanov

**Affiliations:** a Department of Neurosurgery, Hull Royal Infirmary, Hull, United Kingdom; b Department of Neurosurgery, Sheffield Teaching Hospitals NHS Foundation Trust, United Kingdom

**Keywords:** Day-case, Lumbar decompression, Outpatient, Complications

## Abstract

**Introduction:**

Lumbar decompression (LD) surgery, with or without discectomy, is a commonly performed surgical procedure. Despite the concept of day-case LD being reported as early as the 1980s, day-case LD is yet to become routine clinical practice.

**Research question:**

This systematic review aimed to examine the published literature on the safety and complication rates of day-case LD. Secondary outcome measures, including the economic impact and patient satisfaction of day-case LD, were also examined.

**Materials and methods:**

A systematic electronic search was carried out on PubMed, EMBASE and the Cochrane Library between 1999 and January 2022. Studies were screened against predefined inclusion/exclusion criteria with the quality of included studies subsequently being assessed.

**Results:**

In total, 15 studies were included in this review. The majority of studies were undertaken in the USA (n ​= ​8, 53%) and were of a case series design (n ​= ​9, 60%). Reported complication rates ranged from 0% to 7.8%, with nine studies reporting a complication rate of <4%. Readmission rates ranged from 0% to 7.7%. Seven studies quoted a readmission rate of 0%. Five studies found cost saving benefits of day-case LD in comparison to inpatient LD of up to $27,984 (USD). Patient acceptability of day-case LD was consistently high across the six studies that assessed it.

**Discussion and conclusion:**

Day-case LD surgery is a safe and economically efficient surgical option in appropriately selected patients.

## Highlights

• Complication and readmission rates amongst patients undergoing day-case lumbar decompression surgery are low.
• Patient acceptability of day case lumbar decompression surgery is high.
• Day case lumbar decompression surgery is associated with significant cost savings.
• Obstacles to day case lumbar decompression surgery include patient, primary care and ward factors.

## 1 Introduction

Lumbar decompression (LD), with or without discectomy, is one of the most commonly performed spinal surgeries worldwide (Safaee et al., 2021). Given its degenerative nature, the demand for LD surgery is set to increase with ageing populations and increasing public awareness of favorable outcomes (Katz and Harris, 2008; Deyo et al., 2010; Deyo and Mirza, 2006; Helseth et al., 2015). Traditionally LD has been performed on an inpatient basis (Lang et al., 2014), with patients typically staying overnight postoperatively so as to facilitate a period of optimal analgesia and neurological monitoring, thereby mitigating risks of perioperative morbidity.

Increasing pressure on healthcare systems, particularly following the COVID-19 pandemic, has resulted in efforts to better streamline surgical services to improve patient flow and economic efficiency. Same-day discharge models of care provide a true outpatient surgical service, thereby eliminating the costs associated with overnight hospital stays and freeing up resources, as well as minimizing contact with other patients and thereby reducing the risk of developing COVID-19 infection, which has a subsequent impact on perioperative morbidity and mortality.

Whilst the concept same-day discharge following LD was first reported in 1987 by Griffith et al. (Griffith and Marks, 1987), it is yet to become routine clinical practice (Hutton, 2019). Despite same-day discharge following spinal surgery becoming increasingly popular, particularly in the United States (US) (Best and Sasso, 2006; Kurd et al., 2015; Pendharkar et al., 2018)^–^(Best and Sasso, 2006; Kurd et al., 2015; Pendharkar et al., 2018), there remain concerns over its safety, particularly with regards to management of early postoperative complications (Helseth et al., 2015).

In 2019, the reported median length of stay (LOS) following posterior LD in the United Kingdom (UK) was 36 ​h (Hutton, 2019). There is a growing body of level 3–4 evidence to support the safety, efficacy and patient acceptability of day-case spinal surgery (Helseth et al., 2015; Lang et al., 2014; Hutton, 2019; Best and Sasso, 2006, 2007; Sivaganesan et al., 2018; Pugely et al., 2013). On this basis, collating and disseminating evidence on best practice in reducing length of stay and support early discharge was formally recommended (recommendation #15) in the 2019 UK GIRFT Spinal Services Specialty Report (Hutton, 2019).

Whilst several reviews have sought to collate and present the growing burden of evidence supporting the safety and feasibility of day-case LD surgery (Kurd et al., 2015; Pendharkar et al., 2018; Sivaganesan et al., 2018), there remains a paucity of level 1 evidence to date. In 2018, Sivaganesan et al. performed a systematic review of the safety of ambulatory spinal surgery (including cervical and thoracic procedures), which identified nine studies reporting perioperative morbidity following outpatient LD (Sivaganesan et al., 2018). Whilst they found level 3 and level 4 evidence to support the safety and efficacy of outpatient LD, the review was broad, only the PubMed database was searched and the quality of the studies included was not assessed.

The primary aim of this systematic review was to examine the published literature on the safety and complication rates of day-case LD. Secondary outcome measures, including the economic impact and patient satisfaction of day-case LD, were examined where possible.

## 2 Materials and Methods

A systematic review was conducted in accordance with the Preferred Reporting Items for Systematic Reviews and Meta-Analyses (PRISMA) guidelines (Page et al., 2021). The review protocol was registered with the PROSPERO database (CRD42022301978; University of York, Heslington, York, England), the international prospective registry of systematic reviews (NIHR, 2022). The protocol is freely available online (Goacher et al., 2022). No amendments have been made since registration. Outpatient surgery was defined as LD surgery with discharge occurring on the same calendar day.

### 2.1 Search strategy

A systematic electronic search was carried out on the following databases: PubMed (National Library of Medicine, Maryland, USA), EMBASE (Elsevier, Amsterdam, Netherlands) and the Cochrane Library (Wiley, London, England) between 1999 and January 2022. Included studies underwent manual reference searching (forward and backward citation tracking) using PubMed and Google Scholar (Google Inc, California, USA). Trial registries were not searched.

### 2.2 Article screening

Two independent reviewers (EG and MS) screened the titles and abstracts of all search results. In cases of disagreement, a third, independent, reviewer (MI) was consulted. Full texts of included articles were then retrieved and reviewed. The following inclusion criteria were used to screen for eligibility: human studies, English language, full text availability, adult patients (aged 18 years or over), lumbar decompression (+/− discectomy) is a focus of the article, same-day discharge is a focus of the article and the article discusses outcomes of same-day discharge. Case reports, review articles, conference abstracts and articles focused on cervical and/or thoracic spinal surgery with no lumbar spine surgery were excluded from the review. Both randomised and non-randomised studies were included in the review.

### 2.3 Data extraction

Data were extracted from studies by the primary researcher (EG). Extracted data was then independently assessed by a second reviewer (MS). Postoperative complications, where stated with sufficient granularity, were recorded and classified as per the Spinal Adverse Events Severity System, version 2 (SAVES-V2) criteria (Rampersaud et al., 2016). Intra-operative complications, such as dural tears, were not included as these were felt to be independent of planned discharged pathway and thus beyond the scope of this review. A qualitative summary of the associated economic impact and patient satisfaction of day-case surgery was extracted from those studies that examined it.

### 2.4 Quality assessment

Risk of bias was assessed using the Newcastle-Ottawa Scale (Wells, 2015) (NOS) for non-randomised studies (including case-control and cohort studies). The Cochrane Risk of Bias 2 (Higgins et al., 2011) tool was used to assess risk of bias for randomised control trials.

### 2.5 Statistical analysis

The studies included in this review demonstrated significant heterogeneity and therefore a meta-analysis was not justified (Higgins et al., 2021). A narrative, qualitative review of the studies is provided.

## 3 Results

**Fig. 1:**
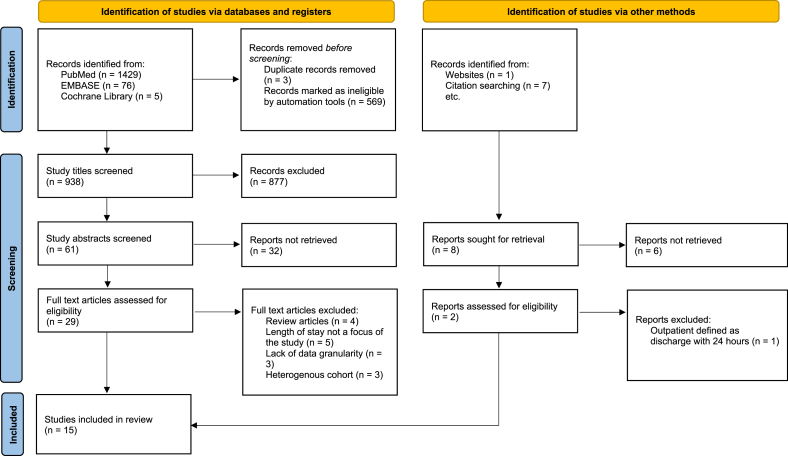
PRISMA flow diagram of study screening process (Page et al., 2021).

In total, 15 studies were included in this review. An initial search of the three electronic databases identified 1507 studies. Following duplicate removal and applied limits, 938 studies were screened (Fig. 1), of which 3.1% (n ​= ​29) underwent full text retrieval and review. Fifteen studies were deemed to have met the inclusion criteria and underwent quality assessment and data extraction. There were no instances of disagreement between reviewers.

### 3.1 Study characteristics

The 15 studies consisted of nine case series, three cohort studies, one randomised control trial (RCT), one case control study and one propensity matching study (Table 1). The majority of studies were undertaken in the US (n ​= ​8, 53%). Number of cases ranged from 13 to 1652. Confirmed complication rates were extrapolated from 11 of the 15 studies (Table 2). Readmission rates were obtained from 14 studies. Six studies assessed patient satisfaction, whilst five studies assessed the economic impact of day-case LD (Table 3).

**Table 1:** Table of included studies. Level of evidenced assessed and defined according to the Oxford Centre for Evidence-based Medicine (CEBM) Criteria (van den Akker et al., 2011).

Study	Year	Country	Study design	Level of evidence	No. of same-day cases	Length of follow up
Abou-Zeid et al. (Scanlon and Richards, 2004)	2014	United Kingdom	Case series	4	36 (+14 within 24 ​h)	6 months
An et al. (An and Simpson, 1999)	1999	United States	Case series	4	61	12 months
Bednar (Gonzalez-Castro et al., 2002)	1999	Canada	Case series	4	121	6 weeks
Best et al. (Best and Sasso, 2007)	2007	United States	Case series	4	233	18 months
Best et al. (Best and Sasso, 2006)	2006	United States	Case series	4	1322	4 years (mean)
Debono et al. (Abou-Zeid et al., 2014)	2017	France	Case series	4	201	6 months
Gonzalez-Castro et al. (Singhal and Bernstein, 2002)	2002	United Kingdom	Randomised control trial	2	13	6 months
Helseth et al. (Helseth et al., 2015)	2015	Norway	Case series	4	1072	12 months
Hirsch et al. (Bednar, 1999)	2019	United States	Case-control	3	35	–
Lang et al. (Lang et al., 2014)	2014	United States	Retrospective cohort	3	183	6 weeks
Pugely et al. (Pugely et al., 2013)	2013	United States	Propensity matching	3	1652	30 days
Safaee et al. (Safaee et al., 2021)	2021	United States	Retrospective cohort	3	152	30 days
Scanlon et al. (Yen and Albargi, 2017)	2004	United States	Case series	4	27	1 month
Singhal et al. (Debono et al., 2017)	2002	Canada	Case series	4	116	6 weeks
Yen et al. (Malik et al., 2020)	2017	Canada	Retrospective cohort	3	25	6 weeks

**Table 2:** Primary outcomes of included studies.

Study	No. of day-cases	Readmission rates (timespan)	Post-operative complication rates (timespan)	Reoperation rates (timespan)
Abou-Zeid et al. (Scanlon and Richards, 2004)	36 (+14 within 24 ​h)	0%	N ​= ​2 – not stated which cohort	0%
An et al. (An and Simpson, 1999)	61	0%	0%	0%
Bednar (Gonzalez-Castro et al., 2002)	121	0%	1.7% (unknown)	0.8% (6 months)
Best et al. (2007) (Best and Sasso, 2007)	233	0.4%, n ​= ​1 (7 days)	1.7% (7 days)	Not reported
Best et al. (2006) (Best and Sasso, 2006)	1322	0.5%, n ​= ​6 (24 ​h)	7.3% (unknown)	Not reported
Debono et al. (Abou-Zeid et al., 2014)	201	1.0%, n ​= ​2 (6 months)	2.0% (6 weeks)	0.5% (6 months)
Gonzalez-Castro et al. (Singhal and Bernstein, 2002)	13	7.7%, n ​= ​1 (7 days)	7.7% (7 days)	Not reported
Helseth et al. (Helseth et al., 2015)	1072	1.8%, n ​= ​19 (41 days)	2.5% (103 days)	6.0% (12 months)
Hirsch et al. (Bednar, 1999)	35	0%	2.9% (unknown)	8.6%
Lang et al. (Lang et al., 2014)	183	5.5%, n ​= ​10 (30 days)	Not quoted	Not reported
Pugely et al. (Pugely et al., 2013)	1652	Not reported	3.5% (unknown)	1.8%
Safaee et al. (Safaee et al., 2021)	152	1.4%, n ​= ​2 (30 days)	Not reported	Not reported
Scanlon et al. (Yen and Albargi, 2017)	27	0% (1 month)	0%	0% (1 month)
Singhal et al. (Debono et al., 2017)	116	0%	1.7% (unknown)	0%
Yen et al. (Malik et al., 2020)	25	0% (6 weeks)	Not reported	Not reported

**Table 3:** Secondary outcomes.

Study	Patient satisfaction	Economic impact (per patient)
An et al. (An and Simpson, 1999)	98.3% satisfied with the experience	∼$2000 USD
Best et al. (2007) (Best and Sasso, 2007)	72.4% would repeat their outpatient procedure	Not assessed
Best et al. (2006) (Best and Sasso, 2006)	81.6% would undergo the procedure again as an outpatient	Not assessed
Debono et al. (Abou-Zeid et al., 2014)	90.5% would recommend the procedure. 81% were either satisfied or very satisfied.	Outpatient costs - €224.08 (EUR) Inpatient costs - €520.38
Gonzalez-Castro et al. (Singhal and Bernstein, 2002)	84.6% felt the day-case procedure appropriate. 15.4% felt it was too short.	Not assessed
Safaee et al. (Safaee et al., 2021)	Not assessed	Same-day (total cost - USD): Teaching hospital - $10,228 Outpatient hospital - $11,348 Overnight (total cost): Teaching hospital - $13,673 Outpatient hospital - $18,680 Admission (total cost): Teaching hospital - $27,984
Scanlon et al. (Yen and Albargi, 2017)	89% rated the experience as either excellent or very good.	Total saving of $4126.67 (USD)
Singhal et al. (Debono et al., 2017)	Not assessed	Total saving of $1440 (CDN)

### 3.2 Quality assessment

A tabulated display of study quality assessment is shown in Table 4. Highest quality was consistently seen in the study selection domain. Non-randomised studies were assessed using the Newcastle-Ottawa quality assessment scale (Wells, 2015). Randomised control trials were assessed using the Cochrane risk-of-bias tool for randomised trials (Higgins et al., 2011).

**Table 4:**
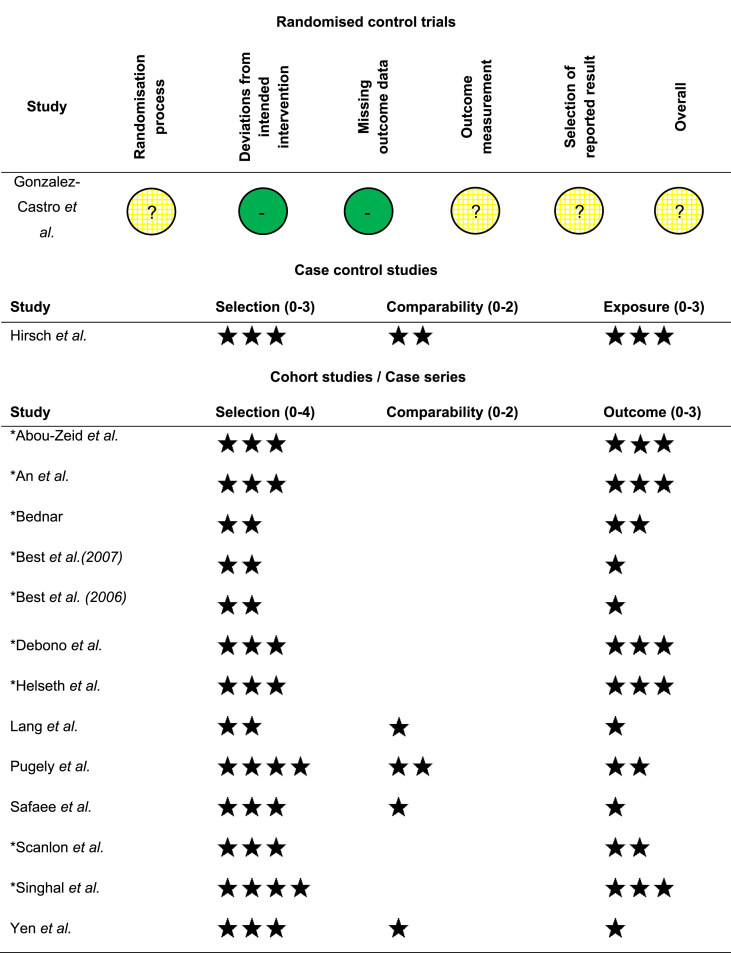
Quality assessment of included studies. Non-randomised studies were assessed using the Newcastle-Ottowa quality assessment scale (Wells, 2015). Randomised control trials were assessed using the Cochrane Risk-of-bias tool for randomised trials (Higgins et al., 2011). ∗denotes case series studies with no comparison. Key: low risk of bias, ? some concerns.

### 3.3 Complications

Postoperative complication rate by study is shown in Table 2. Confirmed complication rates were extrapolated from 11 studies. Reported rates ranged from 0% to 7.8%. Two studies reported a postoperative complication rate of 0% (Scanlon and Richards, 2004; An and Simpson, 1999). Highest complication rates were seen by Gonzalez-Castro et al. (2002) (7.7%, n ​= ​1) and Best et al. (Best and Sasso, 2006) (7.3%, n ​= ​101). Abou-Zeid et al. reported two complications, however, it was not clear if these were in the day-case cohort, or amongst the 14 patients discharged home within 24-h (Abou-Zeid et al., 2014).

Sufficient granularity was provided in 7 studies to grade the complications as the per SAVES-V2 (Rampersaud et al., 2016) criteria (Table 4). Attempted grading was performed on two additional studies (Best and Sasso, 2006; Abou-Zeid et al., 2014). The first of which, Abou-Zeid et al. (2014), provided granularity to grade the complications, but lacked the granularity to assign the complications to either the day-case or 24-h discharge group. The second, a large case series of 1322 by Best et al. (Best and Sasso, 2006), showed 101 complications (7.3%), of which 84 (83.2%) were disc recurrences presenting at an unknown time period, however, subsequent management of the complications was not stated. A range of grades according to the management options of the stated complications was therefore provided.

Complication grading ranged from grade 1 (does not require treatment and has no adverse effect) to grade 4 (requires invasive or complex treatment and is most likely to have a prolonged [>6 months] adverse effect on outcome). Two instances of a grade 4 complication occurred, both of which were unexplained leg weakness (including one foot drop) following LD surgery (Helseth et al., 2015; Singhal and Bernstein, 2002). The most commonly occurring severity of complication were grade 2 (complication requires minor invasive or simple treatment, no long-term effect) and 3 (complication requires invasive or complex treatment and is most likely to have a temporary [<6 months] effect on outcome), occurring in 18 instances.

### 3.4 Readmission rates

Readmission rates ranged from 0% to 7.7% across the 14 studies where they were quoted. Seven studies quoted a readmission rate of 0% (Sivaganesan et al., 2018; Scanlon and Richards, 2004; An and Simpson, 1999; Abou-Zeid et al., 2014; Singhal and Bernstein, 2002; Bednar, 1999; Yen and Albargi, 2017). Highest readmission rates were seen in an RCT (which included only 13 cases of day-case LD) by Gonzalez-Castro et al. (n ​= ​1, 7.8%) (Gonzalez-Castro et al., 2002). Lang et al. reported a readmission rate of 5.5% (n ​= ​10) from a cohort of 183 patients (Lang et al., 2014). The most common reason quoted for readmission was pain. Amongst the nine studies that quoted a timespan for readmission rates, the timespan ranged from 24-h to six months.

### 3.5 Patient satisfaction

Six studies examined patient satisfaction following day-case LD (Best and Sasso, 2006, 2007; Scanlon and Richards, 2004; An and Simpson, 1999; Gonzalez-Castro et al., 2002; Debono et al., 2017). Scanlon et al. showed high levels of patient satisfaction, with 89% rating the experience as either ‘excellent’ or ‘very good’ (Scanlon and Richards, 2004). Debono et al. demonstrated high levels of patient satisfaction associated with day-case LD at two time points (Debono et al., 2017). At six months follow up, 81% were either ‘very satisfied’ or ‘satisfied’ with their experience. This had dropped from 87.5% at day 45. A sentiment that was similarly high, yet decreased over time, was evident in those who would recommend the procedure to a friend (95% at day 45 vs. 90.5% at six months).

### 3.6 Economic impact

Five studies examined the economic impact of day-case LD in comparison to inpatient LD (Safaee et al., 2021; Scanlon and Richards, 2004; An and Simpson, 1999; Singhal and Bernstein, 2002; Debono et al., 2017). All five studies found cost saving benefits of day-case LD (Table 3). Greatest cost difference was observed by Safaee et al., who observed an average total cost of $10,228 (USD) for day-case LD, in comparison to $27,984 for inpatient LD. Care pathway and LOS were quoted as being the main drivers behind this cost difference (Safaee et al., 2021). In addition to their own retrospective cohort study, Safaee et al. performed a systematic review of associated costs of variants of LD surgery (Safaee et al., 2021). Four studies were included in their review (Malik et al., 2020; van den Akker et al., 2011; Parker et al., 2013; Cahill et al., 2013). The review findings were in keeping with their own observations of care pathway and LOS being the primary determinants of cost.

## 4 Discussion and conclusion

Whilst there have been multiple studies documenting the successful implementation of day-case LD, this is the first review to systematically interrogate and assimilate these studies according to the predefined PRISMA (Preferred Reporting Items for Systematic Reviews and Meta-Analysis) guidelines (Page et al., 2021). Low complication and readmission rates were seen across the included studies, with the majority being conducted in North America. Patient acceptability of day-case LD was high, with consistent economic benefits shown for health care providers. Few studies compared day-case LD directly with inpatient LD, with only one small RCT identified. Heterogeneity was seen amongst surgical technique, number of levels decompressed, study design and outcome reporting.

Reported rates of complications following LD (with or without discectomy) range from 3.6% to 12.2% in published studies (Chen et al., 2019; Smith et al., 2010; Epstein, 2018; Rajamani et al., 2021; Li et al., 2008). The highest day-case LD complication rate of any study within this review was 7.7% (Gonzalez-Castro et al., 2002), seen in a small RCT in which one out of 13 patients suffered a complication. This was a SAVES-V2 (Rampersaud et al., 2016) grade 1 (does not require treatment and has no adverse effect). Nine of the 11 studies reporting complication rates reported a day-case complication rate of <4%. Unfortunately, only three of the included studies had an inpatient comparison group (Helseth et al., 2015; Pugely et al., 2013; Gonzalez-Castro et al., 2002). Rates of complications of inpatient LD and day-case LD were similar in each. This may, in part, be explained by the appropriate selection of same-day LD cases. The majority of studies carefully selected patients based on ASA grade (1 or 2)/comorbidities, complexity of surgery, type of surgery (revision or primary) and body mass index (BMI). It is this careful selection of patients that will help to reduce the likelihood of unplanned readmission postoperatively. Unfortunately, there was not sufficient granularity amongst the studies to determine what factors most likely impact rates of readmission. Whilst the authors can infer that patient factors such as high ASA grade, significant comorbidities, poor baseline functional status and obesity play a role in increasing the risk of unplanned readmission, future prospective analyses and quality improvement programmes investigating same-day LD should aim to clarify this selection paradigm so as to facilitate the inclusion of appropriate patients.

All four studies that assessed the economic impact of day-case LD demonstrated lower costs in comparison to inpatient LD (Safaee et al., 2021; Scanlon and Richards, 2004; Singhal and Bernstein, 2002; Debono et al., 2017). Importantly, it should be noted that these reduced costs were seen across three different countries with varying health care models, including both private and public sectors. Given the increasing financial pressures health care providers are facing, the prospect of economically efficient day-case LD surgery is becoming is ever more appealing.

A challenge in establishing homogeneity amongst studies is the definition of ‘outpatient’ surgery. For the purposes of this review, outpatient surgery was classified any LD surgery with discharge occurring on the same calendar day. Some authors and institutions, however, classify outpatient procedures as LD with discharge occurring within 24 ​h of surgery. Such variance in definitions and time-scales is a major impact that weakens the reliability of ‘big data’ sources. Such issues were acknowledged by Gray et al. in 2006 during their interrogation of United States health care databases, including the National Hospital Discharge Survey (NHDS) and the National Survey of Ambulatory Surgery (NSAS), to establish changing population trends in LD surgery (Gray et al., 2006).

Unfortunately, some studies lacked the data granularity to be included in this review. A relatively large cohort study by Purzner et al. in 2011 was excluded despite describing 586 successful day-case spinal surgeries out of a cohort of 602 patients (Purzner et al., 2011). The exact composition of cervical, thoracic and lumbar surgeries in this cohort was unavailable to the authors and thus the study was excluded. It should, however, be noted that the majority (88% of the 602) were listed for LD (Purzner et al., 2011). Additionally, only one of the 602 patients was admitted as an inpatient due to patient preference, suggesting a high acceptability of the concept of day-case LD amongst patients. Results from studies assessing acceptability amongst patients included in this review complement this finding of a high acceptability rate (Scanlon and Richards, 2004; Gonzalez-Castro et al., 2002; Debono et al., 2017).

Whilst the safety profile of day-case LD appears to be acceptable for appropriately selected patients, barriers to day-case LD surgery are well documented and must be addressed if the incidence of day-case LD is to increase. Patient barriers include anxiety and concerns as to post-operative analgesia provisions (Gonzalez-Castro et al., 2002; Ghosh and Sallam, 1994). Similarly, general practioners have expressed concerns, specifically over postoperative complications and analgesia provision (Barrow et al., 1994). Gonzalez-Castro et al. also found barriers amongst clinical staff, finding patients were more likely to be discharged on the day of surgery if they were on a day-case ward, as opposed to an inpatient ward (Gonzalez-Castro et al., 2002). This observation was also identified in the 2019 Spinal Surgery GIRFT report (Hutton, 2019). Not only is it important that patients are appropriately counselled preoperatively, but attention should also be given to ensuring fellow clinical staff are appropriately informed and prepared if day-case LD is to become more widely implemented.

This systematic review found level 3 and level 4 evidence to suggest that day-case LD is safe and feasible in appropriately selected patients. Complication and readmission rates were low, with favorable patient acceptability and economic impacts. Given the increasing economic and waiting list pressures, day-case LD surgery is an increasingly attractive strategy for health care providers. This, coupled with the increasing volume of published literature to support its safety and feasibility, is likely to increase its utilisation in modern day spinal surgery.

## Previous presentations

No prior presentations to disclose.

## Declaration of competing interest

None of the authors declare any conflicts of interest.

No financial funding was received for this study.

## Appendix 1

Search strategies for PubMed, EMBASE and the Cochrane Central Register of Controlled Trials.

(lumbar[Title/Abstract] OR spinal[Title/Abstract] OR spine[Title/Abstract]) AND (decompression[Title/Abstract] OR discectomy[Title/Abstract] OR laminectomy[Title/Abstract] OR microdiscectomy[Title/Abstract] OR lamin∗[Title/Abstract]) AND (discharge[Title/Abstract] OR day case[Title/Abstract] OR outpatient[Title/Abstract] OR inpatient[Title/Abstract] OR same-day[Title/Abstract] OR same day[Title/Abstract] OR length of stay[Title/Abstract])

Limits applied: English language, Human studies, full text available.
